# *Plectranthiasahiahiata*, a new species of perchlet from a mesophotic ecosystem at Rapa Nui (Easter Island) (Teleostei, Serranidae, Anthiadinae)

**DOI:** 10.3897/zookeys.762.24618

**Published:** 2018-05-31

**Authors:** Bart Shepherd, Tyler Phelps, Hudson T. Pinheiro, Alejandro Pérez-Matus, Luiz A. Rocha

**Affiliations:** 1 California Academy of Sciences, San Francisco, CA 94118 USA California Academy of Sciences San Francisco United States of America; 2 Subtidal Ecology Laboratory, Estación Costera de Investigaciones Marinas, Facultad de Ciencias Biologicas, Pontificia Universidad Católica de Chile, Santiago, Chile Pontificia Universidad Católica de Chile Santiago Chile

**Keywords:** endemism, ichthyology, reef fish, South Pacific, taxonomy

## Abstract

A new species of the perchlet genus *Plectranthias* is herein described from a single specimen found at Rapa Nui (Easter Island) in the South Pacific. *Plectranthiasahiahiata* sp. n. was collected at a depth of 83 m in a mesophotic coral ecosystem at Rapa Nui. The main difference between *Plectranthiasahiahiata* and other members of the genus is higher fin-ray counts (X, 18 dorsal; 18 pectoral) and its distinctive coloration. Compared to the three other known eastern South Pacific species, *P.ahiahiata* has more dorsal-fin rays, more pectoral-fin rays, fewer tubed lateral-line scales, fewer gill rakers, a longer head relative to SL, a very short first dorsal spine relative to SL, and a short third anal spine relative to SL. *Plectranthiasahiahiata* is distinguished from western Pacific species, by having more dorsal- and pectoral-fin rays. The closest relative based on genetic divergence (with 12.3% uncorrected divergence in the mitochondrial COI gene) is *Plectranthiaswinniensis*, a widely distributed species, suggesting important links between Rapa Nui and western Pacific islands. This new species adds to the high endemism of the Rapa Nui ichthyofauna, and is further evidence of the importance of mesophotic reefs as unique communities.

## Introduction

Rapa Nui (Easter Island) is the most isolated inhabited island in the Indo-Pacific, located approximately 3,700 km west of Chile and 2,000 km from the nearest inhabited island, Pitcairn (Randall and Cea 2010; Easton et al. 2017). Due to the isolation, the fish fauna exhibits the highest degree of local and regional endemism in both shallow-water and deep-sea fishes in the Indo-Pacific, and very low overall diversity (Friedlander et al. 2013; Easton et al. 2017). Nearly 22% of the shore fishes of Easter Island are endemic (Randall and Cea 2010; Friedlander et al. 2013). Although ichthyological studies since the 1980’s have greatly expanded the knowledge of fish diversity in this ecoregion, there have been relatively few surveys of fishes at depths greater than 40 m (Friedlander et al. 2013; Wieters et al. 2014; Easton et al. 2017). Recent advances in technical mixed-gas rebreather diving are now allowing scientists to safely document small, active, cryptic, and demersal fishes in ways that have been previously impossible (Pyle 2000; Pyle et al. 2016; Rocha et al. 2017). Using these techniques, our team collected a distinctive new species of *Plectranthias* off Hanga Piko, Rapa Nui.

The anthiadine genus *Plectranthias* Bleeker 1873 currently comprises 56 valid species from tropical and temperate waters in the Atlantic, Pacific, and Indian oceans (Eschmeyer et al. 2017). Most of these fishes are found in relatively deep waters (90-420 m) on hard substrates such as mesophotic coral ecosystems and rocky formations (Allen and Walsh 2015; Gill et al. 2016). Due to their diminutive size and cryptic habits they are not often caught by hook and line or in trawls, so they are poorly represented in museum collections, and most species have been described based on a single or a small number of specimens (Randall 1980; Heemstra and Randall 2009; Bineesh et al. 2014; Allen and Walsh 2015; Gill et al. 2016). The three known eastern Pacific species within the genus are *P.exsul* Heemstra and Anderson 1983, *P.nazcae* Anderson 2008, and *P.parini* Anderson and Randall 1991. A fourth eastern Pacific species, *P.lamillai* Rojas and Pequeño 1998, was proposed, however it was later determined to be a junior synonym of *P.exsul* (Anderson and Baldwin 2002). *Plectranthiasparini* is the only other congener known to occur at Rapa Nui, where it was recorded at depths of 150 m during drop-cam surveys (Friedlander et al. 2013). Here *Plectranthiasahiahiata* sp. n. is described and illustrated from a single specimen collected at Rapa Nui.

## Materials and methods

This species is described from a single specimen collected with hand nets while diving on mixed-gas, closed-circuit rebreather (Hollis Prism 2). Counts were performed with a microscope, and morphological characters were measured to the nearest 0.01 mm with digital calipers following the conventions described in Anderson and Heemstra (2012) and Williams et al. (2013). Comparative material included *Plectranthiasjaponicus* Steindachner 1883 (CAS33555), *P.sagamiensis* Katayama 1964 (CAS235596), and *P.winniensis* Tyler 1966 (CAS219169), along with recent publications, and keys and revisions to the genus made by Randall (1980, 1996) and Heemstra and Randall (2008). Morphometric and meristic data for the holotype and comparisons with related species are presented in Table 1 and the Supplementary Online Material. The holotype was deposited at the California Academy of Sciences ichthyological collection.

**Table 1.** Morphological counts and proportional measurements of the known *Plectranthias* species from the eastern South Pacific, the closest sequenced relative, *P.winniensis*, and comparative material from the CAS Ichthyology collection. All proportional measurements are given in percent SL.

	*P.ahiahiata* sp. n.	* P. exsul *	* P. nazcae *	* P. parini *	* P. winniensis *	* P. sagamiensis *	* P. japonicus *
	CAS244172	Anderson and Baldwin (2002)	Anderson (2008)	Anderson and Randall (1991)	CAS219169	CAS235596	CAS33555
Standard length [mm]	39.95	37.6–158	115.0	84.7–163.0	25.5	41.6	78.1
**Counts**:							
Dorsal-fin rays	X, 18	X, 15–16	X, 16	X, 16	X, 16	X, 15	X, 15
Anal-fin rays	III, 7	III, 7	III, 7	III, 7	III, 7	III, 7	III, 7
Pectoral-fin rays	18	16–17	16	15–16	16	13	15
Pelvic-fin rays	I, 5	–	I, 5	–	I, 5	I, 5	I, 5
Tubed lateral line scales	31	36–46	40	37–40	18	30	33
Gill rakers (upper + lower)	6+11	7–10 + 18–22	9 + 19	8 + 18–20	6+10	8+14	7+13
Vertebrae (precaudal + caudal)	10+16	10+16	–	–	–	–	–
Circumpeduncular scales	16	20–22	17–18	16–17	12	14	13
**Measurements**:							
Body depth	30.8	30.1–39.1	38.4	39–41	31.7	35.3	36.3
Body width	16.7	14.9–18.6	17.5	–	17.4	19.6	19.3
Head length	43.3	37.0–39.1	38.4	39–41	39.9	36.5	40.7
Snout length	11.0	8.0–12.3	9.7	8–12	7.7	8.5	8.8
Orbit diameter	9.6	8.4–11.2	10.7	9–12	10.8	10.9	9.3
Postorbital of head length	12.9	17.2–20.0	18.6	–	13.7	18.5	22.3
Upper jaw length	16.8	16.0–19.6	18.7	18.9–20.6	20.1	21.0	19.7
Maxilla width	6.3	4.8–6.3	5.5	–	6.6	6.5	5.9
Caudal peduncle depth	12.5	9.8–13.1	10.9	11.7–11.9	12.0	13.1	13.4
Caudal peduncle length	10.4	18.6–22.7	20.7	–	12.8	12.3	13.5
Caudal fin length	24.6	–	damaged	–	26.8	31.3	broken
Pectoral fin length	33.8	30.1–34.3	30.5	33.9–36.7	30.7	32.7	33.9
Pelvic fin length	26.5	22.3–26.1	23.5	25.6–28.9	24.7	28.7	20.5
Dorsal-fin base length	51.4	49.9–52.8	–	–	48.9	51.8	50.8
Length of 1^st^ dorsal spine	4.5	6.2–7.9	6.2	–	4.4	7.0	4.0
Longest dorsal spine length (number)	15.64 (4^th^)	16.3–19.9 (4^th^ or 5^th^)	16.3 (5^th^)	15.3–18.3 (5^th^)	14.40 (4^th^)	18.4 (5^th^)	12.46 (6^th^)
Length of 1^st^ anal-fin spine	7.2	7.6–11.3	7.6	–	6.0	7.4	5.4
Length of 2^nd^ anal-fin spine	19.0	16.0–20.7	16.0	–	15.8	16.9	13.5
Length of 3^rd^ anal-fin spine	13.7	14.7–15.9	14.8	15.1–17.9	12.3	14.5	10.6

Mitochondrial Cytochrome c oxidase subunit I (COI) DNA was sequenced and analyzed for the new species. DNA extraction and PCR amplification of the COI were performed following Weigt et al. (2012) protocols. DNA sequences were compared to the ten *Plectranthias* species available in GenBank (*P.bennetti*Allen and Walsh 2015: KT601636; *P.flammeus* Williams, Delrieu-Trottin and Planes 2013: KC565477–KC565480; *P.fourmanoiri*Randall 1980: KC567662, KC567663; *P.japonicus*: JQ681323, JQ681324; *P.kamii*Randall 1980: KU943548; *P.kelloggi* Jordan and Evermann 1903: KP267643; *P.longimanus* Weber 1913: JF494178; *P.nanus*Randall 1980: JQ432001–JQ432004, KC565481, KC567661; *P.randalli* Fourmanoir and Rivaton 1980: KP267613; *P.winniensis*: KC565482, KC565483). GenBank accession number for the new species is MH025944.

## Taxonomy

### Plectranthias ahiahiata sp. n.

Taxon classificationAnimaliaPerciformesSerranidae

http://zoobank.org/36C66D98-05BE-40F1-ABD7-9684B51D0E65

**Figure 1.**
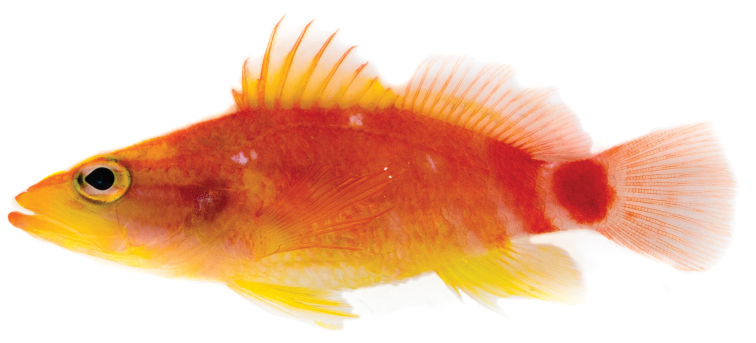
*Plectranthiasahiahiata* sp. n., holotype shortly after death, 39.95 mm SL (photograph: LA Rocha).

Figure 1 , 2 , 3 , Table 1 Sunset perchlet

#### Type locality.

Hanga Piko, Rapa Nui (Easter Island), Chile.

#### Holotype.

**Figure 2.**
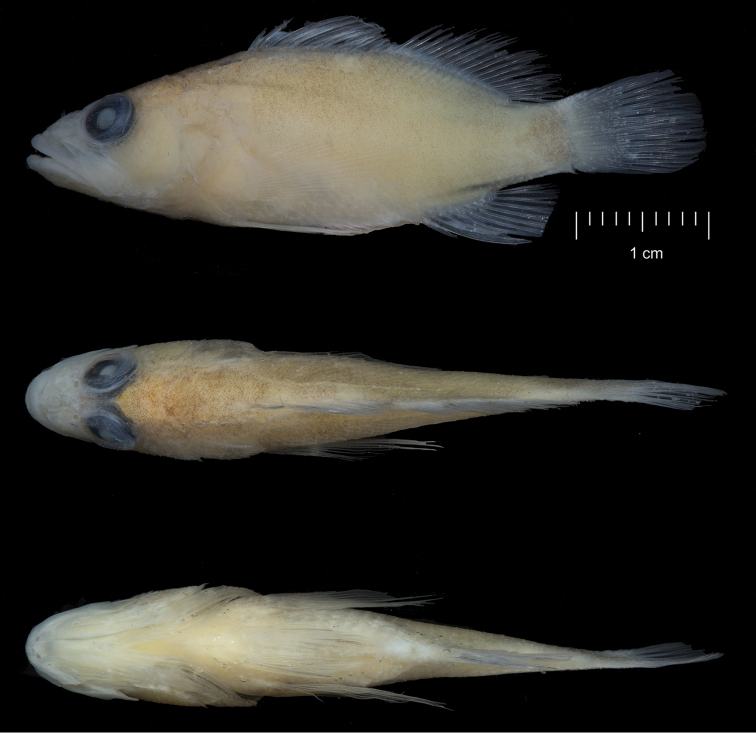
*Plectranthiasahiahiata* sp. n., lateral, dorsal, and ventral views of preserved holotype (photographs: J Fong).

**Figure 3.**
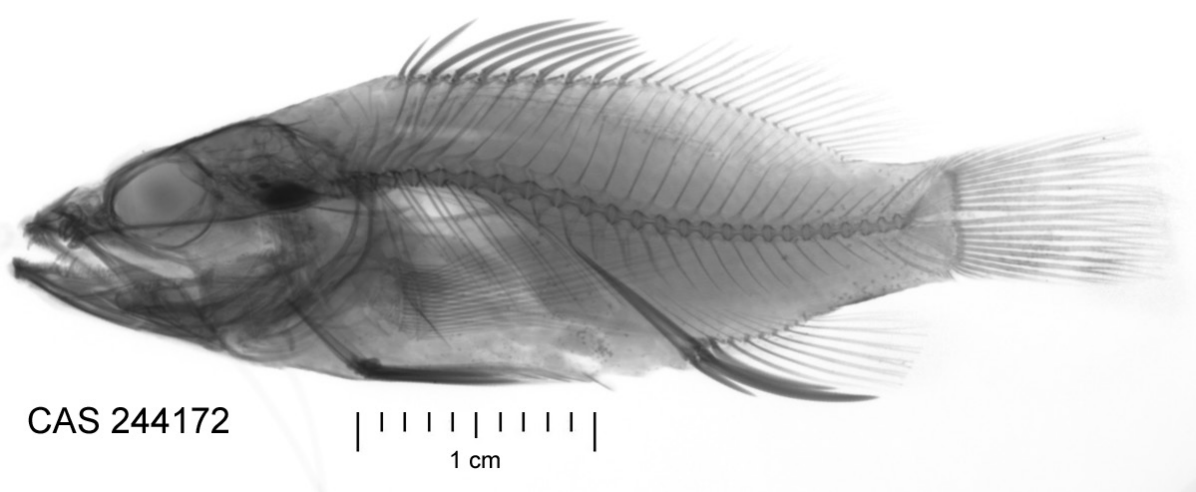
Radiograph of preserved holotype (photograph: J Fong).

CAS244172 (Field number LAR 2644). 39.95 mm SL, GenBank accession number MH025944. Location: Hanga Piko, Rapa Nui, Chile (27°9'12"S 109°26'52"W). Collected by B. Shepherd using a hand-net, 7 March 2017 (Figure 1–3).

#### Comparative material.

Published morphometric and meristic data from the known eastern Pacific species, *Plectranthiasexsul* (Anderson and Baldwin 2002), *P.nazcae* (Anderson 2008) and *P.parini* (Anderson and Randall 1991); the Atlantic species *P.garrupellus*Anderson and Heemstra 2012; the Western Pacific species *P.anthiodes* Gunther 1872, *P.bennetti*, *P.elongatus* Wu, Randall and Chen 2011, *P.flammeus, P.fourmanoiri*, *P.jothyi*Randall 1996, *P.kamii, P. inermis*Randall 1980, *P.randalli*, *P.sheni*Chen and Shao 2002, *P.taylori*Randall 1980, and *P.xanthomaculatus* Wu, Randall and Chen 2011 (Yoshino 1972; Randall 1980; Randall 1996; Wu et al. 2011; Williams et al. 2013; Peristiwady et al. 2014; Allen and Walsh 2015; Tashiro and Motomura 2017); the Japanese species, *P altipinnatus*Katayama and Masuda 1980, *P.takasei* Gill, Tea and Senou 2016, and *P.yamakawai* Yoshino, 1972; and the Arabian species, *P.alcocki* Bineesh, Akhilesh, Gopalakrishnan and Jena 2014. Specimens of *P.japonicus* (CAS33555), *P.sagamiensis* (CAS235596), and *P.winniensis* (CAS19169).

#### Diagnosis.

*Plectranthiasahiahiata* differs from all of its congeners by the following combination of characters: dorsal rays X, 18; pectoral rays 18; longest dorsal spine the fourth; LL continuous and complete with 31 tubed scales; circumpeduncular scales 16; head length 43.3% SL; first dorsal spine 4.5% SL; third anal spine 13.7% SL; gill rakers 6+11; and in coloration: overall orange-red in color, with predominantly yellow snout, dorsal, pelvic and anal fins, a brilliant red spot outlined in white on the caudal peduncle, and four white spots on each side, following the contour of the lateral line.

#### Description.

Proportional measurements and morphological counts of the holotype are given in Table 1. Dorsal rays X, 18; last soft ray branched to base and counted as one; first dorsal spine very short, 4.5% SL; fourth dorsal spine longest, 15.6% SL; dorsal-fin base length 51.4% SL. Anal-fin rays III, 7; last soft ray branched to base and counted as one; anal-fin base 13.7% SL; second anal spine longest at 19.0% SL; anal-fin origin at vertical beneath fifth dorsal-fin ray; pectoral-fin rays 18; length 33.8% SL; pelvic fin I, 5; pelvic-fin length 26.5% SL; pelvic-spine length 16.5% SL; caudal fin with three dorsal and two ventral procurrent rays, two dorsal and one ventral unbranched rays, 7+7 branched rays; tubular lateral-line scales 31; vertical scale rows 30; scales above LL to origin of dorsal fin 3; scales above LL to base of middle dorsal spine 2; scales above LL to origin of anal fin 12; diagonal rows of scales on cheek 4; scales on top of head extending anteriorly to vertical from posterior margin of orbit, where two rows of mid-dorsal scales continue anteriorly to posterior margin of iris; area on top of head between eyes scaleless; no scales on chin, maxilla, or snout; circumpeduncular scales 16; caudal-peduncle length 10.4% SL; caudal-peduncle depth 12.5% SL; body moderately elongate, laterally compressed; body depth 30.8% SL; body width 16.7% SL; gill rakers 6+11; vertebrae 10+16; supraneurals 3.

Mouth large and terminal, slightly upturned; lower jaw protrudes slightly; maxilla expanded posteriorly, extending to below the posterior edge of eye; head long, length 43.3% SL; dorsal profile of head almost straight; post-orbital head length 12.9% SL; snout length 11.0% SL; orbit diameter 9.6% SL; upper jaw with a pair fixed, stout canines on either side of symphysis; inner canine larger of the pair; upper canines flanked internally by villiform band with four to eight rows of depressible, smaller, sharp-tipped teeth; inner rows become progressively longer, innermost row with largest teeth, some larger than upper canines; lower jaw has outer row of fixed, short stout canines at symphysis followed by smaller, depressible, sharp-tipped conical teeth in a villiform band of approx. four to six rows; lower teeth become progressively longer on inner rows, teeth of inner row approx. three times longer than teeth of middle rows, villiform band narrows to one row toward sides of lower jaw; two large fixed canines at midpoint on either side of lower jaw; vomer roughly V-shaped band of two rows of similarly-sized, sharp-tipped, conical teeth; palatines with two rows of small, sharp-tipped conical teeth; tongue small, narrow, pointed, and without teeth.

Scales ctenoid; lateral line complete and broadly arched over pectoral fin following body contour; 31 tubed scales, the last seven in a straight line. Opercle with three spines; preopercle with nine small spines along posterior margin and two antrorse spines on ventral margin; interopercle with one spine; subopercle smooth, with one spine; anterior nostrils positioned at middle of snout, each with a small rounded flap rising from anterior rim; posterior nostrils an elliptical opening at anterior border of orbit.

#### Color in life.

*Body*: overall yellow, orange, and red in color; chest and belly mostly yellow, with yellow extending dorsally to just behind origin of pectoral fin; ventral-most portion of belly white; dorsal portion of body orange-red; series of two to three indistinct orange bars alternating with light pink to white interspaces on the posterior half of the body, followed by a red bar originating below the eleventh dorsal ray and continuing to the base of caudal peduncle; brilliant red spot on the caudal peduncle, approximately same diameter as orbit, outlined in white; four irregularly-shaped white spots on each side of body: first on operculum outlined with dark pink to red border, second just behind the posterior edge of the operculum and beneath the anterior portion of the lateral line and the first and second dorsal spines, third below the eighth dorsal-fin spine, and fourth just below the lateral line beneath and between the fourth and fifth soft dorsal fin rays. These white spots resemble those of *P.winniensis* collected at Pitcairn Island (Randall, unpublished photograph) *Head*: snout, throat, and maxilla mostly yellow; anterior portion of lower lip orange; three indistinct yellow stripes radiating from the anterior portion of the snout through the eye and across the operculum with two light pink to white interspaces; upper interspace extending from lower third of iris to white spot on operculum, lower interspace originating beneath orbit and extending to origin of pectoral fin along upper edge of maxilla; orange stripe originating at tip of snout proceeding across dorsal third of eye and ending at the origin of the lateral line; iris alternating yellow and white lateral stripes, separated with faint, thin red borders; edge of iris outlined in dark-grey to black; *Fins*: spinous portion of dorsal fin translucent yellow; dorsal spines one to six outlined in orange-red along entire length; dorsal spines seven to ten mostly yellow, outlined with red primarily on spine tips; dorsal rays orange-red; lower third of soft dorsal fin mostly yellow, upper two-thirds translucent; caudal-fin membranes translucent with orange-red fin rays; pelvic and anal fins mostly yellow with white and translucent fin rays; pectoral fins translucent yellow with rays outlined in red; base of pectoral fins bright yellow.

#### Color in alcohol.

Light tan overall, with no visible markings (Figure 2).

#### Etymology.

*Plectranthiasahiahiata* is given a Rapa Nui name; the phrase ahiahi-ata means “the last moments of light before nightfall.” The species was given this name because the colors of the fish remind us of the beautiful Rapa Nui sunsets. To be treated as a noun in apposition.

#### Distribution and habitat.

*Plectranthiasahiahiata* is currently only known to occur at Rapa Nui (Easter Island). This fish was collected with hand nets at a depth of 83 m in a rocky patch reef surrounded by a large sandy area, and transported to the surface alive in a perforated plastic jar.

## Discussion

The generic classification of the Anthiadiniae is problematic, and the genus *Plectranthias* is especially in need of revision, as it is not currently defined on the basis of synapomorphies, and there is high variation within many of the defining characters (Anderson and Heemstra 2012; Gill et al. 2016). *Plectranthiasahiahiata* is placed within the genus *Plectranthias* due to the combination of the following characters: dorsal fin X, 18; dorsal fin deeply notched before soft rayed part; anal fin III, 7; second anal spine the longest; pectoral fin rays 18; pelvic fin I, 5; body elongate, oval to oblong; body depth approximately 30-45% of SL; benthic, cryptic habits.

However, placement is provisional, as ongoing genetic and morphological studies indicate *Plectranthias* is not monophyletic, and it is likely that future work will split the genus (Anderson and Heemstra 2012; Gill, et al. 2016). The results of our molecular analysis indicate 12.3% uncorrected divergence in the mitochondrial COI gene from *P.winniensis*, its closest relative among the sequenced members of the genus (Table 2). *Plectranthias* species are minimally represented in GenBank, with COI sequences only available for ten species. Since there are 56 valid species in the genus, presenting a phylogenetic tree with just ten species (less than 20% taxon coverage) could be misleading (Zwickl and Hillis 2002); therefore, a phylogenetic analysis was not performed with the limited available data. As more discoveries are made and more species are sampled for phylogenetics, we can begin to unravel the taxonomic confusion within this genus.

**Table 2.** Uncorrected percent pairwise genetic distances at the mtDNA COI gene between species of *Plectranthias* available in GenBank. *Plectranthias* sp. is an unidentified species from the Gambier Islands (KC567661).

	1	2	3	4	5	6	7	8	9	10	11
1. *P.ahiahiata*	–										
2. *P.bennetti*	19.11	–									
3. *P.flammeus*	20.65	16.38	–								
4. *P.fourmanoiri*	16.89	15.36	17.41	–							
5. *P.japonicus*	17.92	14.68	15.87	13.48	–						
6. *P.kamii*	17.42	17.6	18.91	18.54	15.17	–					
7. *P.kelloggi*	17.75	14.51	15.7	13.31	0.17	14.98	–				
8. *P.longimanus*	17.75	18.09	14.51	18.77	18.94	19.29	18.77	–			
9. *P.nanus*	18.43	17.92	12.97	18.09	18.6	18.73	18.43	16.55	–		
10. *P.randalli*	16.04	18.26	19.62	17.41	16.21	17.79	16.04	19.45	19.97	–	
11. *Plectranthias* sp.	18.6	17.75	12.8	18.26	18.43	18.54	18.26	16.38	0.17	20.14	–
12. *P.winniensis*	12.29	18.09	17.06	16.38	18.09	18.16	17.92	17.58	17.58	17.92	17.41

**Figure 4.**
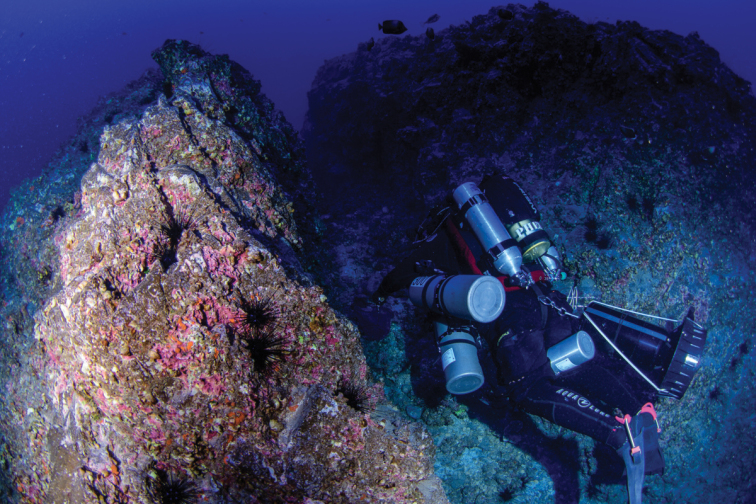
Collection site of the holotype 83 m depth, Hanga Piko, Rapa Nui (photograph: LA Rocha).

Due to the high degree of endemism among shore fishes of Rapa Nui, as well as the large number of known *Plectranthias* species from the western Pacific, it is likely that *P.ahiahiata* is also a locally or regionally endemic species. The fish was found at mesophotic depths on a rocky outcrop with a stand of the stony coral *Leptoserisscabra* (Figure 4). The ambient seawater temperature was approximately 19°C at the time of the dive (7 March 2017). Other fishes at this site included two unknown species of the family Serranidae and one from Pomacentridae, which our group will be describing. Although it is possible that the other species described from the Nazca and Sala y Gómez ridge region, *P.exsul* and *P.nazcae*, are geographically sympatric, the only member of the genus previously documented from Rapa Nui is *P.parini*. The affinity with *P.winniensis*, a widespread species ranging across the Indo-Pacific from the Red Sea to Pitcairn, suggests important links between the ichthyofauna of Rapa Nui and the western Pacific islands.

Mesophotic ecosystems are known to shelter numerous specialist and exclusive species, which contribute to making this environment biologically unique in tropical and semitropical environments (Pinheiro et al. 2016). The few surveys of fishes at depths greater than 40 m at Rapa Nui (Easton et al. 2017) and the high degree of endemism at the island suggest that many other unknown species remain to be discovered. Scientists’ use of closed-circuit, mixed-gas rebreathers is allowing exploration and comparison between mesophotic and shallow coral ecosystems. The many new species descriptions resulting from the exploration of mesophotic reefs reveal the importance and uniqueness of these ecosystems (Pyle et al. 2016; Baldwin et al. 2016, 2018; Rocha et al. 2017).

## Supplementary Material

XML Treatment for Plectranthias ahiahiata
